# Higher age at diagnosis of hemochromatosis is the strongest predictor of the occurrence of hepatocellular carcinoma in the Swiss hemochromatosis cohort: A prospective longitudinal observational study

**DOI:** 10.1097/MD.0000000000012886

**Published:** 2018-10-19

**Authors:** Albina Nowak, Rebekka S. Giger, Pierre-Alexandre Krayenbuehl

**Affiliations:** a Department of Endocrinology and Clinical Nutrition, University Hospital Zurich; b Department of Internal Medicine, Psychiatric University Hospital Zurich, University of Zurich; c Department of Internal Medicine, University Hospital Zurich, Zurich; d Department of Internal Medicine, Linth Hospital, Uznach, Switzerland.

**Keywords:** hemochromatosis, hepatocellular carcinoma, risk factor

## Abstract

Hereditary hemochromatosis (HH) is the most common genetic disease in Caucasians which is characterized by an increased intestinal iron absorption, resulting into a progressive accumulation of iron in organs including liver, heart, and pancreas, leading to their progressive dysfunction. Hepatocellular carcinoma (HCC) is a long-term complication of HH, which contributes to increased mortality.

We evaluated the risk factors of HCC in a prospective cohort of Swiss hemochromatosis patients with a long-term follow-up.

We included 147 patients with the mean age at diagnosis of 48 years, in whom 70% were men. Overall, 9% of the patients developed HCC during the mean follow-up time of 14 years (range 1–40 years). Patients with HCC had higher age at diagnosis (61 ± 11 vs 47 ± 13 years, *P* = .003), more frequently liver cirrhosis on biopsy (90% vs 37.5%, *P* = .004), and higher serum ferritin levels [3704 (Q1:2025, Q3:4463) vs 1338 (Q1:691, Q3:2468) μg/L, *P* < .001], they needed more iron removed by phlebotomy until its depletion [8.9 (Q1:7.2, Q3:10.1) vs 3.8 (Q1:1.6, Q3:8.9) g, *P* = .029], compared to non-HCC patients. After adjustment for possible confounders, only higher age at diagnosis remained significantly associated with HCC development (odds ratio 1.19, 95% CI 0.056–0.397, *P* = .001, per year).

Higher age at diagnosis showed the strongest association with the occurrence of HCC in Swiss hemochromatosis patients. Patients who were diagnosed at a higher age and with a high iron overload (serum ferritin levels >1000 μg/L) require regular screening even if they have no liver cirrhosis.

## 1 Introduction

Hereditary hemochromatosis (HH), an autosomal recessive disease, is the most common inborn disease in Caucasians, particularly in those with Nordic and Celtic ancestry, with the prevalence of homozygosity of approximately 0.5% and of heterozygosity about 10%.^[1–4]^ The most frequent causative mutation is a substitution of tyrosine for cysteine at position 282 of the high iron Fe (HFE) protein product (C282Y); the second frequent mutation is a substitution of aspartate for histidine at position 63 (H63D).^[5,6]^

HH is characterized by an increased intestinal iron absorption, which results into a progressive accumulation of iron in organs including liver, heart, and pancreas, leading to their progressive dysfunction.^[3,7]^ Hepcidin, the most important regulator of systemic iron metabolism, is influenced by body iron stores, erythropoietic activity, and hypoxia, and plays the dominant role in controlling iron absorption. Its serum levels are decreased in HFE hemochromatosis.^[8]^

Hepatocellular carcinoma (HCC) is a dreaded long-term complication of HH, as it largely contributes to the increased mortality in patients with HH.^[9]^ Its occurrence has been associated with a high iron overload and shown to be mostly a consequence of homozygosity of the *C282Y* mutation.^[10]^ The major pathogenic mechanism of HCC development is the oxidative deoxyribonucleic acid (DNA) damage, which is induced by the formation of hydroxyl radicals due to the catalytic activity of iron.^[11]^

To prevent this complication, HH patients at risk for HCC need to be monitored more closely and treated more intensively.^[9,12,13]^ However, to identify the risk factors for HCC development and to estimate the prevalence of HCC remains an unmet clinical need, because of controversial reports in previous studies.^[9,12–17]^ In our nationwide long-term study of a large cohort with genetically proven HH patients, who were regularly followed-up at our specialized iron metabolism tertiary care center or in the primary care, we aimed to elucidate these questions.

## 2 Methods

This is an analysis of the prospective Swiss hemochromatosis cohort. The study was conducted in accordance with the principles of the Helsinki Declaration. Approval for this study was obtained from the Ethics Committee of the Canton of Zurich, Switzerland, (KEK 2010-0364). All patients participating in the study gave written informed consent.

### 2.1 Study population and the HH diagnosis

We contacted the patients during the routine clinical visits at the University Hospital Zurich or via the Swiss Hemochromatosis patient's organization and suggested to participate in this study. The patients included in this analysis were diagnosed between 1971 and 2013.

The patients had been initially diagnosed as having HH either due to clinical or biochemical or histological signs of iron overload or through family screening. Iron overload was defined as elevated serum ferritin (>300 μg/L for men, >200 μg/L for women) and/or elevated transferrin saturation (>50% for men, >45% for women). The diagnosis of HH was genetically confirmed in all patients: *C282Y* homozygotes, *C282Y*/H63D compound heterozygotes or H63D homozygotes.

These consecutive HH patients were registered into the Swiss Hemochromatosis database, included into the prospective cohort and were offered routine examinations including a clinical assessment, laboratory blood analyses, and annual liver imaging using ultrasound, computed tomography scan, or magnetic resonance imaging (MRI).

The following features were considered clinical manifestations of HH: metacarpophalangeal (MCP) arthropathy, diabetes mellitus, hepatopathy, cardiomyopathy, and hypogonadism. The presence of arthropathy was assessed by history and clinical examination. Hepatopathy was defined as either elevated liver enzymes (aspartate and/or alanine transaminase above the upper limit of normal as indicated by the corresponding laboratory) or positive liver histology. Positive liver histology was defined as portal-portal bridging fibrosis or cirrhosis, corresponding to grade 3 and 4 on a scoring system from 0 to 4.^[18,19]^ Cardiomyopathy was specified as either dilated or restrictive cardiomyopathy with documented cardiac iron overload. The diagnosis of hypogonadism was based on typical clinical characteristics in combination with low testosterone levels.

### 2.2 Data assessment

The following data were collected at the time of diagnosis of HH and included into the present analysis: type of HFE-gene mutation, age, sex, serum ferritin level, transferrin saturation, elevation of transaminases, histological result of liver biopsy (if available), and serologic testing for hepatitis B and C and clinical manifestation.

The following variables were recorded during the course of the disease and extracted from the medical records for the present analysis: amount of iron removed through phlebotomy until achievement of a serum ferritin level of <300 μg/L (assuming that 500 mL of blood removed correspond to 250 mg of iron), body mass index (BMI), date and result of the most recent liver imaging, and the self-reported amount of alcohol consumption. Considerable alcohol consumption was defined as intake of >20 g (men) or >10 g (women) of ethanol per day.

If patients were followed by primary care physicians, their medical records were obtained for this study.

### 2.3 Treatment and HCC diagnosis

All patients except one—who refused therapy—received treatment with intensive phlebotomies from the time of diagnosis until depletion of iron stores followed by maintenance phlebotomies where necessary.

HCC was suspected on the routine liver imaging and confirmed by biopsy in all patients. Patients without focal hepatic lesions on imaging studies were considered negative for HCC.

### 2.4 Statistical analysis

Statistical analysis was performed using the IBM SPSS statistics 22 software package (34). Variables were compared using the Student *t* test, the Mann-Whitney *U* test, or the Fisher exact test (all 2-tailed). A *P* value of <.05 was considered significant. Multiple logistic regression was used to assess for the independent effects of relevant variables on the outcome. To correct for small sample bias and to deal with the problem of quasicomplete separation, Firth's penalized maximum likelihood estimation (35) was used for the multiple logistic regression analysis performed with *R* (36).

## 3 Results

### 3.1 Main clinical characteristics at the time of diagnosis

**Figure 1:**
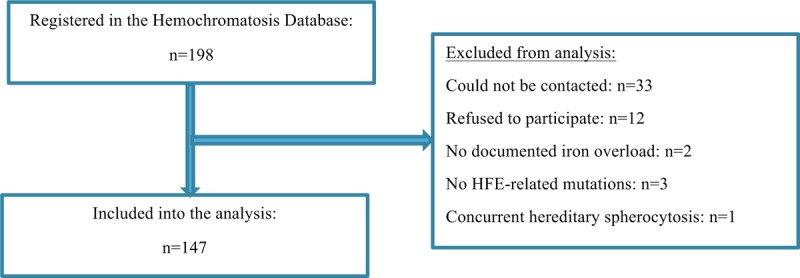
Flow diagram of the Swiss Hemochromatosis Cohort. HFE = high iron Fe.

From 198 patients who were registered in the database, 51 were excluded from the study for various reasons: 12 did not wish to participate, 2 never had a documented iron overload, 3 did not have HFE-related mutations, 1 patient suffered from concurrent hereditary spherocytosis, and 33 patients could not be contacted (Fig. 1). The main clinical and biochemical characteristics at the time of diagnosis of the remaining 147 patients, who were included into the present analysis, are shown in Table 1.

**Table 1:**
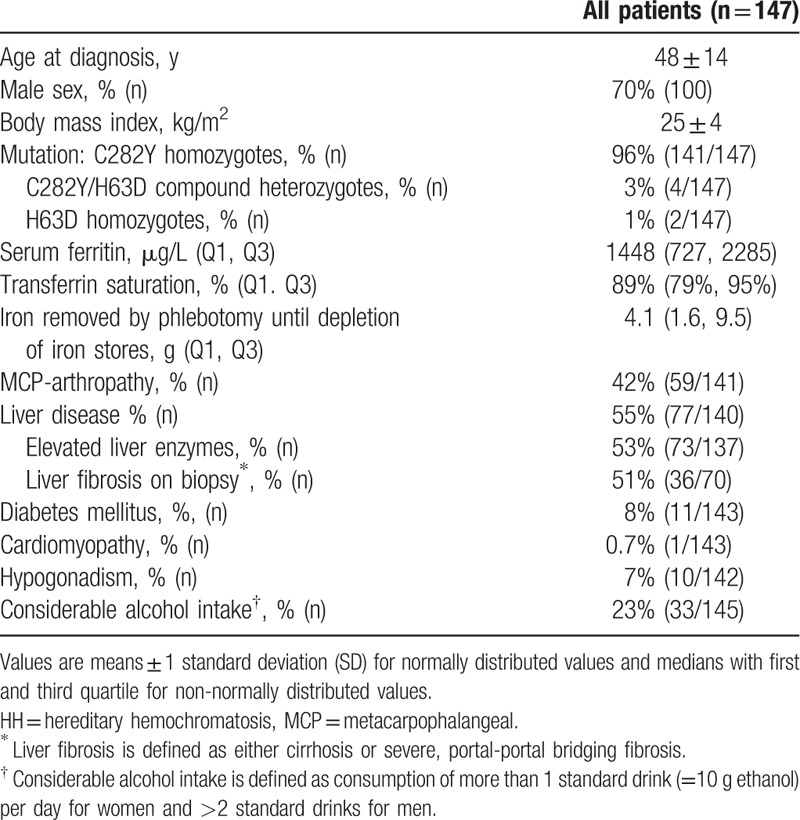
Main clinical and biochemical characteristics at the time of diagnosis of hereditary hemochromatosis.

The mean age at diagnosis of HH was 48 years (range 18–89 years). Seventy percent of the patients were men. The majority of patients (141/147) were *C282Y* homozygotes. Serum ferritin levels ranged from 49 to 9180 μg/L with a median of 1448 μg/L. Transferrin saturation was almost uniformly elevated. Twenty-nine percent of patients did not show any organ-specific clinical manifestations. The most frequent clinical manifestations were MCP arthropathy, prevalent in 59 of 141 patients, and liver disease, in 77 of 140 patients. Liver disease was mostly manifest in the form of elevated liver enzymes. Overall, 70 patients underwent liver biopsy, which was indicated by the treating physicians independent of this study; 36 of biopsies showed severe fibrosis or cirrhosis. Diabetes mellitus was present in 8%, hypogonadism in 7%, and cardiomyopathy in 1% of the patients.

### 3.2 HCC prevalence and clinical characteristics of the HCC patients

The mean length of the follow-up was 14 years (range 1–40 years). Seventeen patients were excluded from this analysis, because they had no liver imaging, 13 because they had deceased and no information about their cause of death was available.

Ten patients were diagnosed with HCC, resulting in an overall prevalence of 9%. All 10 patients were men and affected by a homozygous *C282Y* mutation. None of them suffered from concurrent chronic viral hepatitis. All 10 showed an MCP arthropathy and elevated liver enzymes at the time of diagnosis of HH. One patient had a normal liver biopsy, whereas the other 9 suffered from severe fibrosis or cirrhosis. The time span from diagnosis of HH to detection of HCC ranged from 0 to 30 years.

We further analyzed the prevalence of HCC among subgroups stratified according to 2 of the most important risk factors—ferritin and liver cirrhosis.^[20,21]^ Of patients with a serum ferritin level >1000 μg/L at the time of diagnosis of HH, 14% (10/69) developed HCC. When analyzing only patients with a serum ferritin level higher than 2000 μg/L, the resulting prevalence was 21% (7/34). Of those patients with positive liver histology, 33% (9/27) were eventually diagnosed with HCC.

### 3.3 Risk factors for hepatocellular carcinoma

To determine the risk factors, we first compared the main clinical and biochemical characteristics at the time of diagnosis of HH between patients with and without HCC. Table 2 shows this juxtaposition with the statistical significance of the differences based on the univariate analysis. Patients with HCC were significantly older when diagnosed with HH; the youngest patient later suffering from HCC was 40 years old at the time of diagnosis of HH. Serum ferritin levels were significantly higher in HCC patients, ranging from 1329 to 6807 μg/L, as was the amount of iron removed through the phlebotomy, ranging from 7 to 18 g. The year of diagnosis did not differ significantly. MCP arthropathy, elevated liver enzymes, and histological signs of liver cirrhosis were significantly associated with HCC.

**Table 2:**
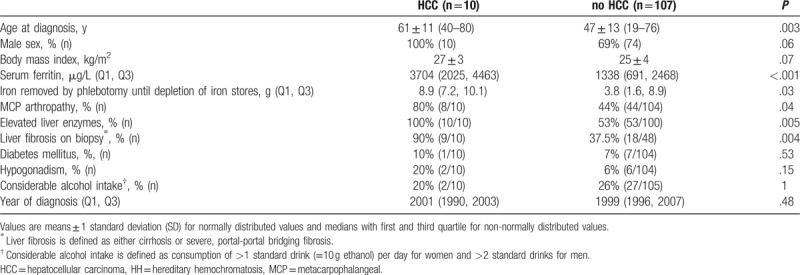
Comparison of main characteristics at the time of diagnosis of hereditary hemochromatosis between patients with and without hepatocellular carcinoma.

Because both, liver cirrhosis and HCC, have been reported to be rare in patients with ferritin levels below 1000 μg/L,^[12,13,20,22,23]^ we compared patients with higher ferritin levels with and without HCC (Table 3) to evaluate possible indicators for HCC in the patients most at risk. Higher age at diagnosis was significantly associated with HCC in the univariate analysis. The other parameters showing a significant association were higher ferritin levels, liver fibrosis on biopsy, and higher BMI. There was no correlation between age and BMI.

**Table 3:**
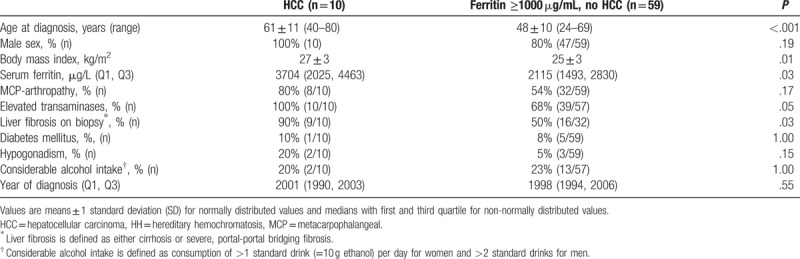
Comparison between patients with hepatocellular carcinoma and patients without hepatocellular carcinoma and serum ferritin >1000 μg/L at the time of diagnosis of hereditary hemochromatosis.

To assess for the independent effect of the most relevant variables associated with HCC, we performed a multiple logistic regression analysis. The results are shown in Table 4. The variables chosen for this analysis include those shown to be significant in Table 3 as well as those found positive in all HCC patients (male sex, MCP arthropathy, elevated liver enzymes), shown in Table 2. We further included considerable alcohol intake, as it has previously been reported to cause liver cirrhosis and HCC.^[24]^ Liver histology was not included in this analysis because of the relatively small number of patients who underwent liver biopsy. As all patients with HCC were men and had elevated liver enzymes and MCP arthropathy, Firth's penalized maximum likelihood estimation^[25]^ was used to deal with the issue of the quasicomplete separation. The only variable still significant in this analysis was age at diagnosis.

**Table 4:**
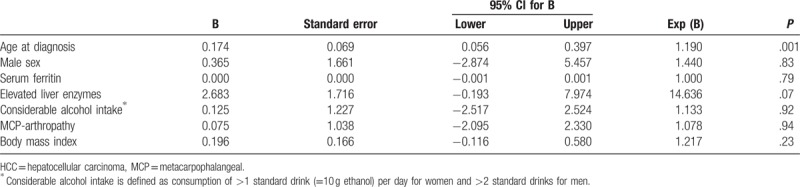
Multiple logistic regression comparing patients with and without hepatocellular carcinoma.

To corroborate the finding of age as an independent risk factor, we executed the same analysis including the results of the liver biopsy. When analyzing all patients with a serum ferritin level above 1000 μg/L who underwent liver biopsy at diagnosis of HH, age at diagnosis was still significantly higher among HCC patients [Exp(B) 1.213, *P* = .007; total of 40 cases were analyzed]. The other variables did not differ significantly. We further compared HCC patients with patients without HCC and very high ferritin levels (>2000 μg/L), which corresponds to a matched population for this variable. Age at diagnosis was still significantly higher among HCC patients (60.5 ± 11 vs 48.4 ± 7.8 years; *P* = .001). Likewise, when including only patients with positive liver histology, HCC patients were significantly older than non-HCC patients (63.5 ± 16 vs 50.0 ± 7 years; *P* = .004).

### 3.4 Other malignancies

During the follow-up time, 14 other malignancies than HCC occurred in 13 hemochromatosis patients: 3 lung cancers, 3 prostate cancers, 2 tonsil cancers, 1 kidney cancer, 1 testicular cancer, 2 melanomas, 1 spinocellular carcinoma, and 1 basalioma. One patient with 2 malignancies had melanoma and basalioma.

Serum ferritin levels at diagnosis of hemochromatosis were not significantly different in patients who developed other malignancies compared with the other patients (2085 ± 2748 vs 1840 ± 1683 μg/L; *P* = .17). In patients with serum ferritin levels above and under 1000 μg/L, other malignancies occurred with a similar frequency (*P* = .32). In the univariate regression analysis, serum ferritin levels at diagnosis were not predictive for the development of other malignancies (odds ratio 1.0; 95% CI 1.00–1.001; *P* = .08).

## 4 Discussion

In this large, well-defined cohort of HH patients, all with genetically proven diagnosis, with a very long follow-up time, we found that HCC, occurring in 9% of all HH patients, is still a prevalent HH complication.

Higher age at diagnosis was the strongest predictor of HCC development in this cohort. It seems to be a surrogate marker for the duration of exposure to iron overload, because progressive accumulation of iron with increasing age is assumed in patients without iron depletion therapies.^[26]^ In our analyses, the high iron overload, expressed by the serum ferritin levels of above 1000 μg/L, lost its significance in the multiple logistic regression analysis after adjustment for age at diagnosis. This finding suggests that the duration of exposure to toxic iron levels may be more important for the development of HCC than the amount of the liver iron burden.

Another risk factor for HCC in this cohort was a high iron overload, reflected by the serum ferritin levels of above 1000 μg/L at diagnosis. There are several pathological and experimental studies providing evidence for the direct carcinogenic effects of iron through oxidative stress and epigenetic DNA, lipid, and protein modifications, which lead to hepatocyte necrosis and apoptosis.^[11,20]^ In addition, the mutagenic radicals, generated by oxidative stress, may suppress the immune response against formation of malignant hepatocytes, either directly or via inflammation pathways.^[20,27]^ Furthermore, iron overload has been shown to cause insulin resistance which can enhance HCC development.^[28]^ This effect was independent of the presence of cirrhosis.^[29–32]^ Also in our cohort, 1 patient who developed HCC had no liver cirrhosis and a normal liver biopsy at baseline.

Some previous studies also found an overall prevalence of HCC of 8% to 10% and a high prevalence among patients with cirrhosis.^[9,13,17,21]^ Interestingly, the prevalence of HCC among HH patients remained similar over the last 40 years.

Importantly, the development of liver cirrhosis has been shown to correlate with the severity of iron overload in previous studies.^[21,33]^ Along the same lines, HCC patients had significantly higher ferritin levels at diagnosis in our and also in previous studies. In contrast to other studies, we could not find a significant association between considerable alcohol consumption and development of HCC. A possible explanation could be the low prevalence of a heavy alcohol intake in our cohort (only 5 patients who consume >300 g of ethanol per week), at least as self-reported.

If HCC is diagnosed at a symptomatic stage, a poor prognosis can be expected, because curative surgical or interventional options are limited.^[34]^ Thus, screening of high-risk populations is recommended to detect HCC at an early stage.^[35,36]^ So far, American Association for the Study of Liver Diseases guidelines recommend regular screening for HCC in patients with liver cirrhosis only.^[35]^ We, however, recommend regular screening also in patients who were diagnosed and initiated on iron depletion therapy at a higher age and had/or a high iron overload (>1000 μg/L) at diagnosis. In these patients, liver ultrasonography at 6-month intervals should be the preferable screening method. In patients with a moderate (<1000 μg/L) iron overload and without cirrhosis, we use to perform liver ultrasonography at 12-month intervals. Screening in noncirrhotic livers can aid in the early diagnosis of HCC in the cases where HCC develops without cirrhosis. Screening intervals in patients with moderate iron overload without cirrhosis can be individually determined in discussion with the patient, depending on availability of other risk factors. Screening should be continued throughout life because HCC can develop many years after the achievement of iron depletion.^[21]^

The biomarker alpha-fetoprotein (AFP) is less helpful for HCC diagnosis because of its low sensitivity and specificity, especially with small sizes of HCC: AFP levels remain normal in up to 40% of patients with HCC, particularly during the early stage of the disease.^[37]^

Interestingly, iron overload was not significantly associated with the occurrence of the other malignancies during the follow-up time in this study. However, this finding is limited because no screening programs for malignancies, other than HCC, are established in hemochromatosis. Thus, a number of other malignancies could have remained undiagnosed.

Several limitations merit consideration: only 70 of 147 patients in the cohort underwent liver biopsies. This is an increasingly common limitation because liver biopsy is no longer needed to assess liver iron content, which nowadays can be done noninvasively by MRI.^[38]^ Similarly, although we saw an association of increased BMI with HCC, which have been previously observed and associated with nonalcoholic fatty liver disease (NAFLD),^[39]^ we cannot make a statement in this regard from our study due to the lack of information about NAFLD in biopsy specimens. Furthermore, no conclusion can be made from our study about the influence of concurrent chronic viral hepatitis, as only 3 patients (all without HCC) suffered from chronic hepatitis B or C.

The strength of the study is that we recruited patients not only from a tertiary care center, but also from the primary care. Thus, we consider the cohort to adequately reflect the clinical picture of HH encountered in daily practice. Furthermore, in this prospective cohort, we applied regular patient assessment, and included the collection of complete clinical, biochemical, genetic, and phenotypic information. These advantages were frequently lacking in other studies. Also, this homogeneous study population was exposed to the same environmental conditions due to medical treatment and geographic and cultural proximities.

## 5 Conclusions

Higher age at diagnosis showed the strongest association with the occurrence of HCC in Swiss hemochromatosis patients. Early diagnosis and therapy initiation benefit the disease course and help to prevent HCC. Patients who were diagnosed having HH at a higher age and with a high iron overload, expressed by the serum ferritin levels of >1000 μg/L, are at increased risk to develop HCC and require regular screening even if they have no liver cirrhosis.

## Author contributions

**Conceptualization:** Albina Nowak, Rebekka S. Giger, Pierre-Alexandre Krayenbuehl.

**Data curation:** Rebekka S. Giger, Pierre-Alexandre Krayenbuehl.

**Formal analysis:** Rebekka S. Giger, Pierre-Alexandre Krayenbuehl.

**Investigation:** Albina Nowak, Rebekka S. Giger, Pierre-Alexandre Krayenbuehl.

**Methodology:** Albina Nowak, Rebekka S. Giger, Pierre-Alexandre Krayenbuehl.

**Project administration:** Rebekka S. Giger.

**Writing – original draft:** Albina Nowak, Rebekka S. Giger, Pierre-Alexandre Krayenbuehl.

**Writing – review and editing:** Albina Nowak, Rebekka S. Giger, Pierre-Alexandre Krayenbuehl.
